# Lipidomic profiling of PEX11β knockout T-REx 293 cells reveals lipid shifts suggesting potential metabolic perturbations

**DOI:** 10.1016/j.bbrep.2026.102801

**Published:** 2026-09-17

**Authors:** Samantha Jeng, Mansha Soni, Vishal C. Kalel, Wolfgang Schliebs, Ralf Erdmann, Frank Schulz

**Affiliations:** a Department of Systems Biochemistry, Institute of Biochemistry and Pathobiochemistry, Faculty of Medicine, Ruhr University Bochum, Universitätsstraße 150, Bochum, 44801, Germany; b Department of Organic Chemistry, Faculty of Chemistry and Biochemistry, Ruhr University Bochum, Universitätsstraße 150, Bochum, 44801, Germany

**Keywords:** Peroxisome biogenesis, Peroxisome biogenesis disorders, Peroxin, PEX11β, Redox sensitivity

## Abstract

Peroxisomes function in reactive oxygen species (ROS) and lipid metabolism. Genetic defects in peroxisome biogenesis cause severe neurological disorders collectively termed peroxisome biogenesis disorders (PBDs). Dysregulated peroxisomes are also increasingly implicated in cancer and therapy resistance. Our comparative analysis of peroxin knockouts (PEX KO) in a human embryonic kidney-derived cell line by untargeted lipidomics reveals distinct lipid fingerprints across PEX KO conditions. PEX3 and PEX14 KO cell lines are highly enriched in very-long-chain fatty acids (VLCFAs) and depleted in ether- and plasmalogen-linked phospholipids. While they differ in the same lipids, PEX3 KO cells demonstrate stronger shifts from the wild-type profile than PEX14 KO cells. In contrast, PEX11β KO cells nearly match the wild-type lipid profile except for several highly enriched lipid analytes, most notably ceramides. These distinct metabolic adaptations are paralleled by the cell lines’ divergent responses to glucose-6-phosphate dehydrogenase (G6PD) inhibition, suggesting PEX KO phenotype-specific differences in redox stress and NADPH source reliance.

## Highlights

• PEX3, PEX11β, and PEX14 KO cells each have distinct lipid profiles.
• PEX11β KO cells are highly enriched in certain ceramide species.
• PEX11β KO cells are more sensitive to G6PD inhibition, while PEX3 KO cells are less susceptible.

## 1 Introduction

Peroxisomes are single-membrane-bound organelles of eukaryotic cells essential for diverse metabolic functions, primarily lipid metabolism (particularly the β-oxidation of very-long-chain fatty acids and ether and plasmalogen phospholipid synthesis) and the detoxification of reactive oxygen species. They originate from the endoplasmic reticulum (ER) and can arise from existing peroxisomes [1], [2]. Their metabolic activity requires various enzymes located in peroxisomes. As peroxisomes lack protein synthesis capabilities, proteins are imported post-translationally through a dedicated import machinery recognizing peroxisomal targeting signals [3], [4]. This import mechanism is unique in its ability to translocate fully folded proteins and, at times, oligomeric protein complexes [5], [6]. Disrupted peroxisomal function results in peroxisomal biogenesis disorders such as Zellweger Spectrum Disorder (ZSD) [7].

The proteins implicated in ZSD are peroxisome biogenesis factors, also known as peroxins (abbreviated PEX). In *Homo sapiens*, peroxin functions include peroxisomal import receptors and the docking complex (PEX5, PEX7, PEX13, PEX14, PEX39), membrane biogenesis (PEX3, PEX16, PEX19), matrix protein translocation and receptor recycling (PEX1, PEX2, PEX6, PEX10, PEX12, PEX26), and peroxisomal proliferation (PEX11 family) [3], [8]. Defects in peroxins result in ZSD or ZSD-like symptoms, such as impaired vision and growth, early mortality, and respiratory problems [7].

Beyond ZSD, the role of peroxisomes is increasingly recognized in oncogenesis, where tumor cells exploit peroxisomal pathways to facilitate progression. Here, altered lipid metabolism may favor cell proliferation, particularly through increased membrane component synthesis. However, the lipidomic shifts associated with specific PEX deficiencies remain to be fully elucidated. Zou et al. [9] demonstrated that PEX3 and PEX14 knockout (KO) ovarian cancer cells are less sensitive to ferroptosis by GPX4 inhibition due to reduced ether phospholipid levels. Abe et al. [10] demonstrated that PEX11β-deficient cells have decreased levels of docosahexaenoic acid (C22:6)-containing phospholipids, while arachidonic acid (C20:4) and oleic acid (C18:1) levels are elevated. Nonetheless, a comparative analysis across PEX KO mutant types is lacking.

This work compares the metabolic fingerprints of PEX3 (biogenesis defect), PEX11β (division defect), and PEX14 (matrix import defect) KO cells through untargeted lipidomics to explore how different types of peroxisomal disruptions impact lipid homeostasis. Further, we examine these defect-specific effects under redox stress. We challenge the selected PEX KO cells with the glucose-6-phosphate (G6PD) inhibitor G6PDi-1, building on previous work linking PEX KO lipid differences to ferroptosis resilience [9]. Thus, we identify PEX KO-specific vulnerabilities in pentose phosphate pathway-mediated redox resilience driven by NADPH antioxidant capacity.

## 2 Materials and methods

### 2.1 Cell culture

Wild-type and PEX KO T-REx 293 (Invitrogen, USA, Cat. No. R71007) cells were grown in complete DMEM (Gibco, UK) containing 25 mM d-glucose and 4 mM l-glutamine with 10% fetal bovine serum, 100,000 U/L penicillin, and 100 mg/L streptomycin at 37 °C with 5% CO_2_. Cells were seeded at a density of 3 × 10^5^ cells per well in quadruplicate in 6-well plates 72 h before harvesting. Cell-free medium controls were pipetted into the same plates.

### 2.2 CRISPR/Cas9-mediated knockout of PEX11β in T-REx 293 cells

Wild-type T-REx 293 cells were co-transfected with a plasmid containing one guide RNA (ACAGAAACAGATTCGACAAC) and another synthetic sgRNA (Synthego) (AAGAGAGCAAGCATACTGGG) using an Amaxa Nucleofector II and Kit V (Lonza Biosciences). Fluorescence-activated single-cell sorting was performed using a MoFlo Astrios EQ (Beckman Coulter) into 96-well plates, which were incubated at 37 °C with 5% CO_2_ before clone selection and genetic screening by TIDE (tracking of indels by decomposition) analysis. The other PEX KO cell lines were generated in prior work [11]. Verification of the knockout was performed by sequencing and functional complementation (Fig. S10, Supplementary Information 1).

### 2.3 Cell processing for mass spectrometry

Cells were washed twice with 2 mL of ice-cold phosphate-buffered saline (PBS). 750 μL of ice-cold LC-MS-grade methanol combined with 625 μL of Milli-Q water was added to the lower zone of each dish. Cells were scraped into the quenching/extraction solution with a rubber scraper. Cells were transferred to pre-cooled conical 10 mL glass centrifuge tubes, snap-frozen with dry ice, and stored at −80 °C.

Cells were freeze-thawed three times at room temperature (RT), resuspended, and refrozen in liquid nitrogen, vortexing at 2500 rpm for 3 min after each freeze-thaw cycle. Subsequently, cells were sonicated for 30 s for complete lysis.

### 2.4 Lipid extraction

Lipids were extracted using a modified Matyash protocol [12]. Analytical-grade methyl *tert*-butyl ether (MTBE) was added to the methanol/water cell suspension. Samples were vortexed for 10 min at 1900 rpm and incubated at RT for 15 min for phase separation. The upper phase was transferred, and the solvent was evaporated on a miVac QUATTRO vacuum concentrator at 30 °C for 1 h. Dried lipid extracts were reconstituted in LC-MS-grade isopropanol and vortexed at 1900 rpm for 20 s. Solutions were stored at −80 °C overnight (14–18 h). Samples were centrifuged at 12,700 rpm (Eppendorf 5810R, S-4-104) at 4 °C for 15 min. 90 μL of supernatant was transferred to HPLC vials with 250-μL glass inserts. A quality control (QC) sample was prepared by pooling 5 μL from each sample.

### 2.5 Data acquisition

Untargeted lipidomics was performed using high-performance liquid chromatography with high-resolution mass spectrometry (HPLC-HRMS). 5-μL aliquots of each sample were injected into a Compact Q-TOF Mass Spectrometer (Bruker Daltonik GmbH) with a standard electrospray ionization source, coupled with an Ultimate 3000 HPLC system (Thermo Fisher Scientific).

Solvent blanks, media blanks and QC samples were injected after every 5th sample to account for technical reproducibility. Further details on parameters and controls for data acquisition can be found in the Supplementary Information.

### 2.6 Data processing

Raw mass spectrometry files were converted to mzML format using MSConvert [13]. The resulting mzML files were corrected for lock-mass recalibration of MS2 spectra [14] and imported into MZmine [15]. Processing steps included mass detection, ADAP chromatogram builder, ^13^C isotope filter, join aligner, and gap filling (Table S1). This was applied to both ion modes, yielding 2187 features in positive ion mode and 1206 features in negative ion mode.

### 2.7 Statistical analysis

The aligned feature lists were exported as *.csv files and imported into MetaboAnalyst [16]. One outlier from each group was removed. Features with constant values across all samples were removed from analysis. Missing values were imputed with 1/5th of the minimum positive value. Normalization was also conducted using the workflow recommended in MetaboAnalyst. Sample normalization was first performed by dividing the peak areas by the corresponding total protein content obtained from cell pellet immunoblots to account for variations in sample biomass and technical variability. Further details on obtaining peak areas can be found in Supplementary Information 1. Data were subsequently log10-transformed to stabilize variance and improve comparability across features, followed by data scaling to reduce the dominance of highly abundant metabolites. Different scaling methods were evaluated, and Pareto scaling was selected as it best preserved the biological variation while minimizing noise amplification (Supplementary Information 3). Principal component analysis (PCA) was performed to assess the reproducibility of the LC-MS analysis and extraction procedure. Significant features were identified using one-way ANOVA followed by Tukey's HSD post hoc test (FDR-adjusted *p* < 0.05) (Supplementary Information 4), while pairwise comparisons were performed using Student's *t*-test (FDR-adjusted *p* < 0.05). Heatmaps were generated using Euclidean distance and average linkage.

### 2.8 Lipid annotation

Lipid annotation was conducted using SIRIUS [17], Global Natural Products Social Molecular Networking (GNPS2) [18], and the MZmine lipid annotation tool [19]. Lipids were annotated following the Lipidomics Standards Initiative (LSI) [20], using a mass tolerance of ±10 ppm. [35] All annotations were based on matching characteristic MS/MS fragment ions to entries in public spectral databases and the GNPS library. For phosphatidylcholine (PC), phosphatidylethanolamine (PE), and sphingomyelin (SM) classes, characteristic headgroup fragments were used for structural confirmation. The fragment ion *m/z* 184.07 in positive ion mode was used to confirm PC and SM, and *m/z* 140.01 in negative ion mode to confirm PE. Annotation was supported by characteristic fatty acyl fragment ions, including acylium ions ([RCO]+) in positive ion mode and carboxylate ([RCOO]^-^) in negative ion mode. For sphingolipid annotation, characteristic fragment ions derived from the sphingoid base backbone were also used [21] (Table S2, Supplementary Information 1). Further information on the identification level and fragment ions can be found in Table S2.

### 2.9 Treatment of PEX KO T-REx293 cells with G6PDi-1

Cells were seeded at 5000 cells/well in 100 μL of medium in a 96-well plate 16–20 h before drug treatment. The medium was then replaced with 100 μL of medium containing a 1.3-fold dilution series of G6PDi-1 (MedChemExpress) in DMSO. For a 100%-signal untreated control, only medium was used, and for a 0%-signal control, 100 μL of medium containing a 1:100 dilution of 100 μM RSL3 (MedChemExpress) in DMSO was used, which killed all cells within 4 h.

After 68 h, 25 μL of 0.15 mg/mL resazurin in Hank's buffered saline solution (Gibco) was added to each well, and plates were re-incubated 4 h at 37 °C with 5% CO_2_ before reading at 530 nm Excitation/585 nm Emission and 530 nm Excitation/575 nm Emission with a BioTek Cytation 5 plate reader. Cell viability was normalized to the average 530 nm/585 nm-530 nm/575 nm differences of the untreated WT cell replicates in each of two separate assays (one assay performed with WT, PEX3 KO, and PEX14 KO cells; second assay with WT and PEX11β KO cells due to logistical constraints). Curves were fit with GraphPad Prism 8.0.2 nonlinear [Inhibitor] vs. normalized response with variable slope.

## 3 Results

### 3.1 Untargeted lipidomics demonstrates PEX KO-specific metabolic fingerprints

LC-MS-based lipidomic analyses of the PEX KO whole cell lysates revealed clear differences between the lipid compositions of PEX3 and PEX14 KO cells compared to those of the WT and PEX11β KO cells.

**Fig. 1:**
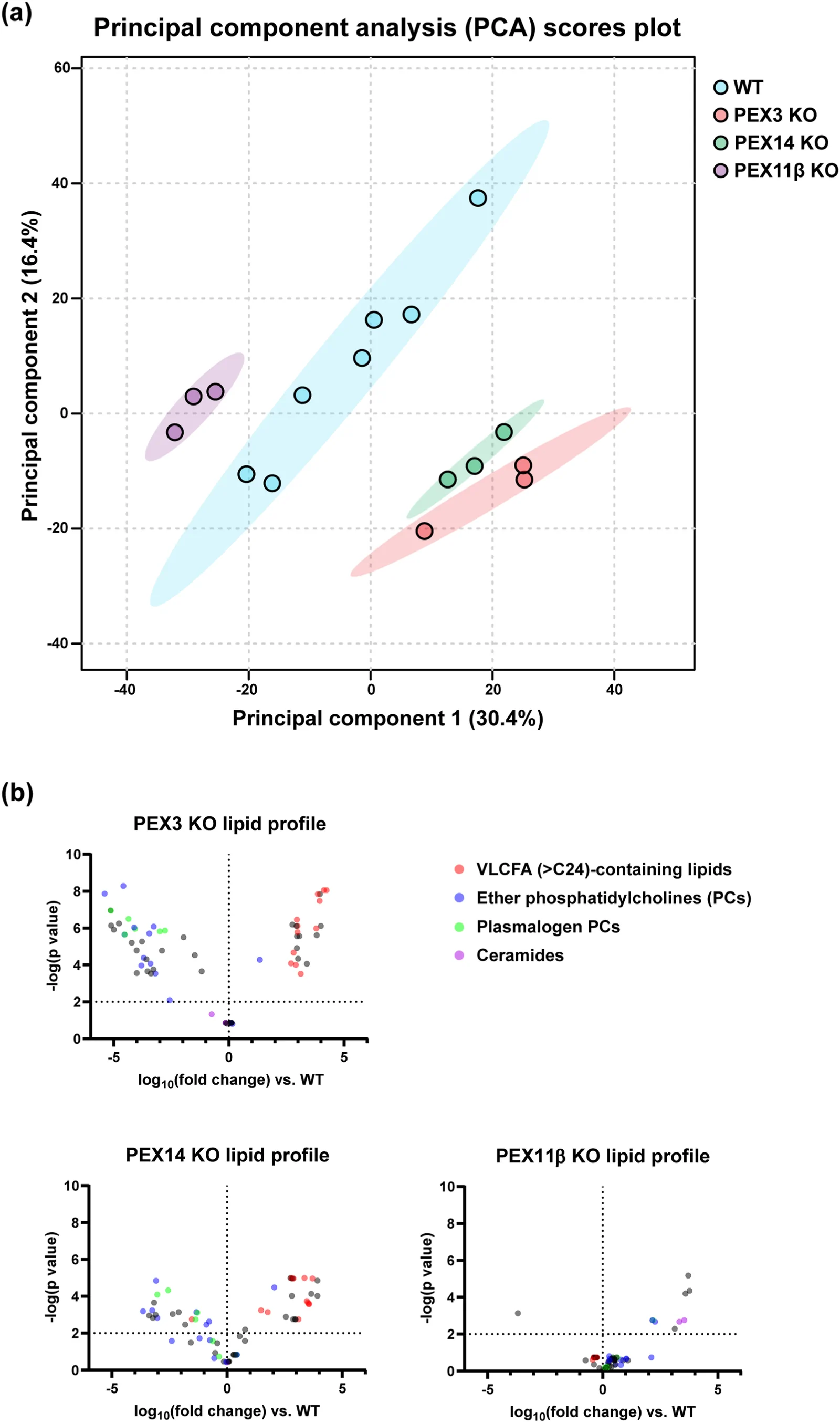
**PEX KO cells demonstrate divergent LC-MS lipid profiles.** **(a)** Principal component analysis (PCA) in positive ion mode shows complete lipidomic separation among all cell lines (n = 7 for WT; n = 3 for all others). **(b)** Volcano plots highlight differences in PEX KO cells from the WT. PEX3 and PEX14 KO cells are lower in ether (blue) and plasmalogen (green) phosphatidylcholines (PCs) and higher in very-long-chain fatty acid-containing lipids (red). PEX11β KO cells are similar to the WT but differ most in two ceramide species (lower right).

PCA was performed to assess data quality, evaluate the clustering of biological replicates, and identify potential outliers. One sample per replicate group was excluded from subsequent analyses to account for out-of-range lock mass noise. The remaining biological replicates clustered closely, indicating analytical reproducibility. Clear separation was observed between the WT and PEX knockout cell lines, with PEX3 KO showing the greatest separation from WT (Fig. 1a). These findings indicate distinct global lipidomic profiles among the cell lines and provided the basis for subsequent univariate analyses.

**Fig. 2:**
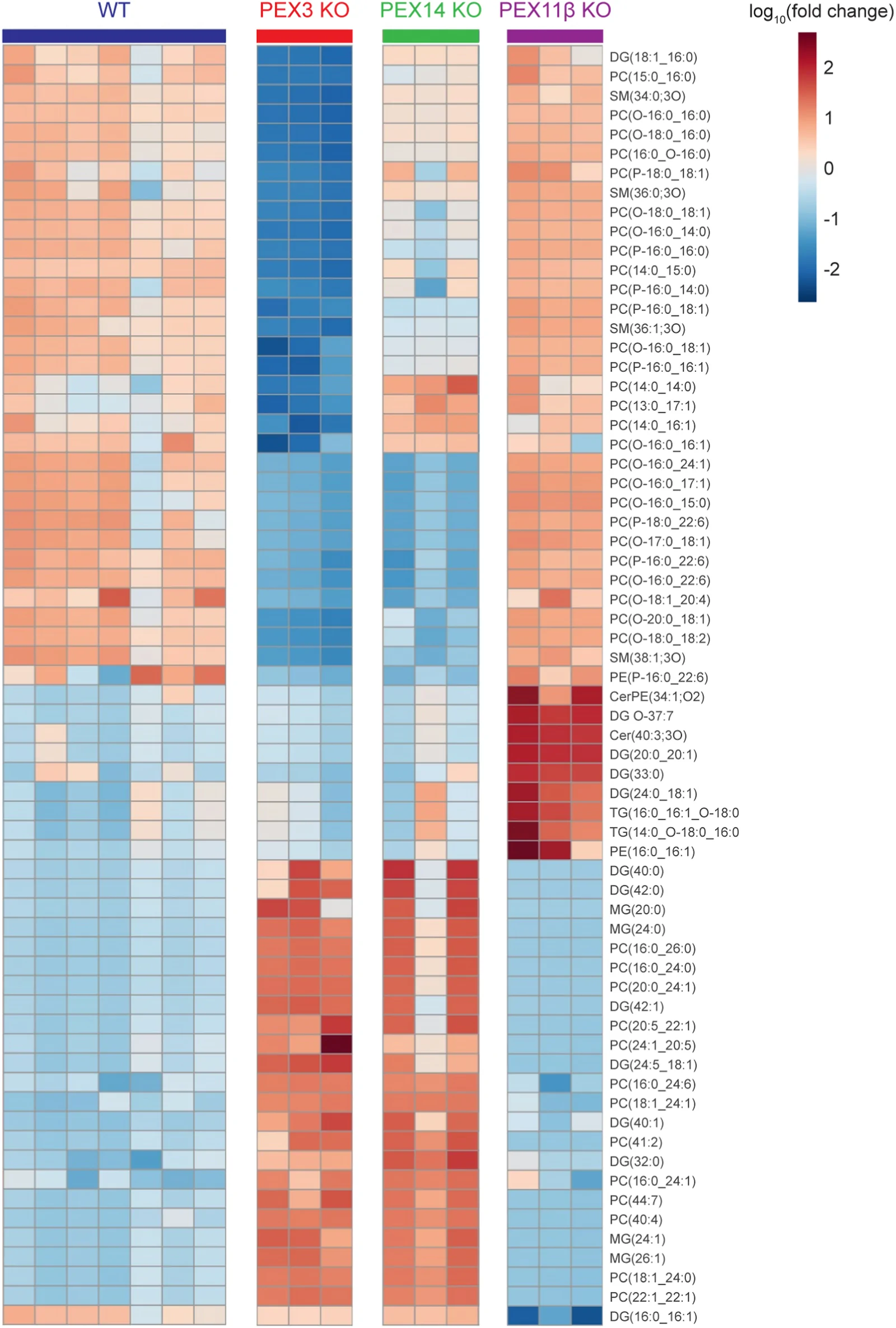
**Heatmap of lipid analytes in T-REx293 WT and PEX KO cell samples.** Relative abundances of lipid analytes (log_10_(fold change), normalized to the average of all samples). Lipid annotations are shown to the right of the heatmap. PEX11β KO (n = 3 biological replicates) and WT (n = 7) cells have similar lipid profiles apart from highly enriched analytes in PEX11β KO (CerPE(34:1, O2) through PE(16:0_16:1)). The PEX3 KO (n = 3) profile differs most from the WT. The lipid profile of PEX14 KO (n = 3) represents an intermediate between PEX3 KO and WT.

ANOVA revealed that approximately 20% of the detected features were significantly altered across the cell lines (FDR-adjusted *p* < 0.05). The heatmap (Fig. 2) highlights the most differentially abundant lipid analytes among the PEX knockout cell lines, while details of the annotated significant features are provided in Table S2 (Supplementary Information 1). Pairwise comparisons between each knockout cell line and WT were subsequently performed (Fig. 1b), where the –log(*p*) values were derived from Student's *t*-tests. While PEX3 and PEX14 KO have similar lipid profiles that differ from the WT, the lipid profile of PEX11β KO differs from the WT in only a few highly enriched or depleted lipids. Further details on the pairwise comparisons among the PEX knockout cell lines are provided in Supplementary Information 1.

PEX3 and PEX14 KO cells differ in many of the same lipid analytes compared to the WT cells, but the degree to which they differ is generally stronger in PEX3 KO. One notable exception is DG(40:1), which is higher in PEX14 KO cells than in PEX3 KO cells, but both are >1000-fold higher in DG(40:1) than WT cells. The lipid analytes that are higher in PEX3 and PEX14 KO cells represent very-long-chain fatty acid (VLCFA)-containing di- and monoacylglycerol (DG and MG) as well as phosphatidylcholine (PC) analytes. The analytes that were lower in PEX3 and PEX14 KO cells largely contain ether or plasmalogen linkages in their fatty acid chains.

The PEX11β KO lipid profile largely resembles the WT lipid profile but differs from it in ways not observed in the PEX3 or PEX14 KO profiles. PEX11β KO cells are higher in certain di- and triacylglycerols than WT cells but much lower in DG(16:0_16:1). The ceramides Cer(40:3; 3O) and CerPE(34:1; O2) are also much higher in PEX11β KO cells, with each relative log_10_(fold change) representing an over-3500-fold difference from the WT cells. The odd-numbered chain phosphatidylcholines PC(13:0_17:1) and PC(14:0_15:0) are also moderately higher in PEX11β KO cells.

Thus, untargeted lipidomic analysis reveals distinct, peroxin-defect-specific lipid signatures across the PEX KO cell lines.

### 3.2 G6PD inhibition reveals divergent redox sensitivities across PEX KO cell lines

**Fig. 3:**
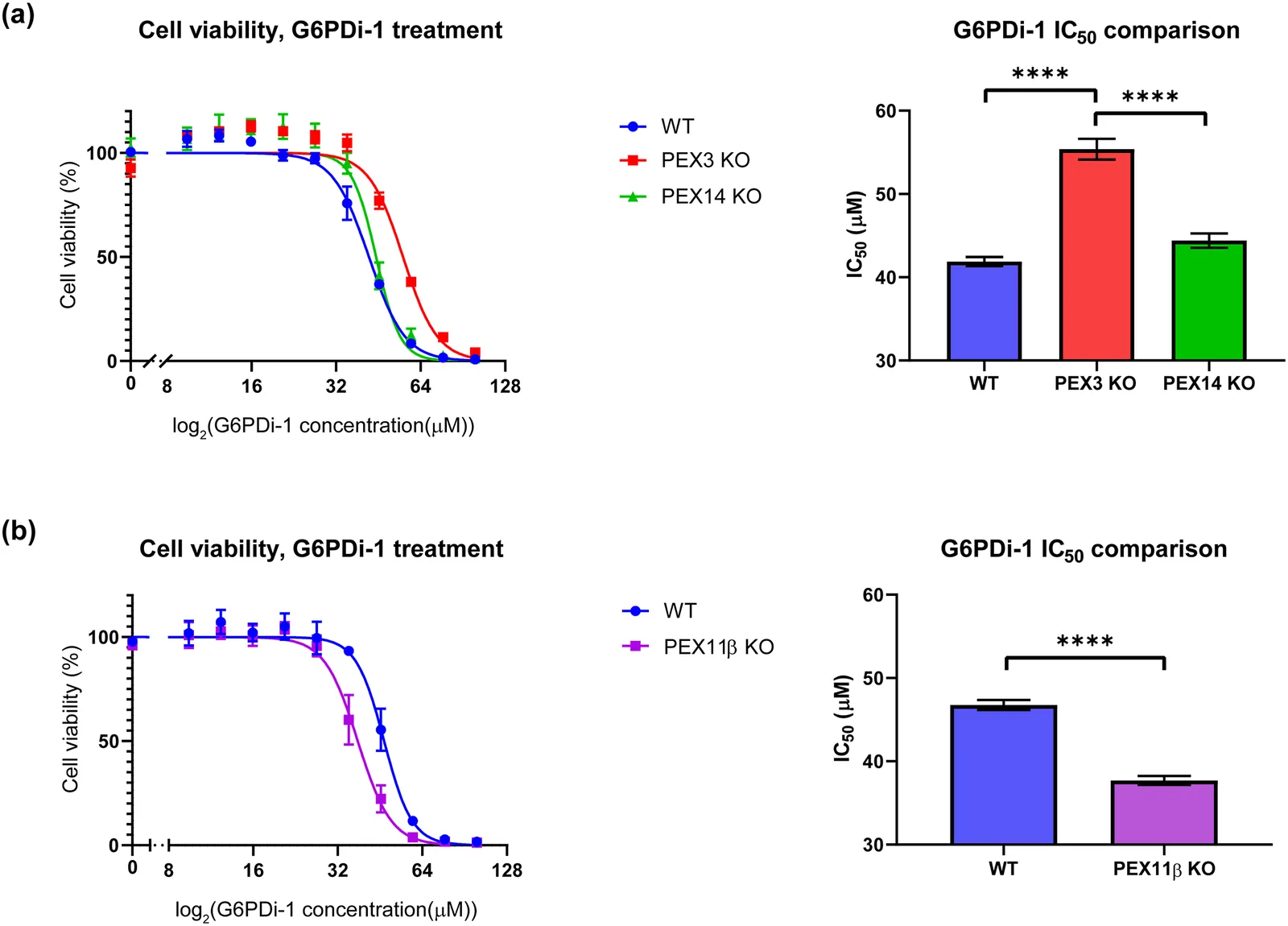
**Effect of G6PDi-1 on T-REx293 WT and PEX KO cell viability.** T-REx 293 cell lines were treated 72 h (n = 3 biological replicates, in two separate batches) with G6PDi-1. PEX3 KO cells **(a)** have increased resistance (p < 0.0001 compared to WT and PEX14 KO) to G6PDi-1-mediated NADPH depletion, while PEX14 KO cells did not differ significantly (p = 0.6332) from WT cells. PEX11β KO cells **(b)** showed increased sensitivity to G6PDi-1 (p < 0.0001).

Upon treatment with the glucose-6-phosphate dehydrogenase inhibitor G6PDi-1, PEX KO cell lines show divergent responses. While PEX3 KO cells are less sensitive to G6PDi-1 than wild-type cells, PEX11β KO cells are more sensitive. No significant difference in G6PDi-1 sensitivity was observed in PEX14 KO cells (Fig. 3).

## 4 Discussion

Untargeted lipidomic analysis demonstrated divergent and cell line-specific lipid profiles across the PEX KO cell lines. PEX3 KO cells were characterized by the starkest shift from the WT profile, with effectively-zero levels of ether and plasmalogen phospholipids and high levels of VLCFA-containing MGs and DGs. Likewise, PEX14 KO cells had reduced and increased levels of the same lipid analytes. This is expected, as intact peroxisomes and peroxisomal matrix import are required for ether and plasmalogen lipid synthesis, as well as the β-oxidation of VLCFAs. Interestingly, four phosphatidylcholine species (PC(14:0_14:0), PC(13:0_17:1), PC(14:0_16:1), and PC(O-16:0_16:1)) were strongly depleted only in the PEX3 KO cells. As PEX3 KO cells contain no peroxisomal membranes, these species may represent constituents of the peroxisomal membrane. The fatty acid chain lengths of 13–17 carbons suggest a thin membrane conducive to peroxisomal function.

Analysis of PEX11β KO cells may hint at not-yet-reported metabolic deficiencies. Abe et al. [10] suggest that cells with other peroxisome fission deficiencies share similar lipid profiles with PEX11β KO cells. They report elevated levels of arachidonic acid (C20:4) and oleic acid (C18:1) in the phospholipids of DRP1-, NME3-, or PEX11β-deficient patient fibroblasts and decreased levels of phospholipids containing docosahexaenoic acid (C22:6). Accordingly, we observe that oleic acid-containing lipid species DG(24:0_18:1) and PE(16:0_18:1) are higher in PEX11β KO cells than WT cells, with log_10_(fold change) values of 2.160 and 2.116, respectively.

We also observe the enrichment of the ceramides Cer(40:3; 3O) and CerPE(34:1; O2) and ether-linked triacylglycerols (TGs) TG(13:0_O-18:0_16:0) and TG(16:0_16:1_O-18:0) in PEX11β KO cells. These accumulated lipid analytes hint at possible lipotoxicity-driven dysregulation [22]. Further, it was observed in *Saccharomyces cerevisiae* that ablation of orthologous Pex11p reduces the rate of β-oxidation at similar fatty acid chain lengths [23]. Whether such a lipid dysregulation in PEX11β KO cells is driven by a compensatory shift in sphingolipid metabolism or a disruption in ER-peroxisome contact sites is unknown.

The accumulation of the observed ceramides may be associated with the redox imbalance observed in the treatment of PEX11β KO cells with G6PDi-1, although no predicted correlation between the PEX11β KO lipidome and ceramide pathways is present (LION analysis, Supplementary Information 1). Further probing with redox balance, metabolomics, and enzyme kinetics assays is required. Ceramide synthesis from free fatty acids requires NADPH, as demonstrated by Singh [24]. Further, ceramides are involved in signaling NADH/NADPH oxidase regulation by homocysteine, affecting superoxide (O_2_^−^) concentrations in cells [25].

Oddly, PEX3 KO cells are more resistant to G6PDi-1-mediated NADPH depletion than WT cells, while PEX11β KO cells are less resistant. PEX3 KO cells may therefore potentially adapt to their significant redox stress by metabolic reprogramming. This could lead to the broad upregulation of NADPH sources, including alternative sources such as isocitrate dehydrogenase 1 (IDH1) or malate dehydrogenase. Indeed, IDH1 appears to be upregulated (log_2_(fold change) = 0.88, p = 0.004) in PEX3 KO HEK293T cells in work by Segev-Nakar and Koren [26], although this difference may not reflect broad metabolic reprogramming, as there was no evidence of G6PD expression changes (log_2_(fold change) = 0.42, p = 0.205). Nevertheless, PEX3 KO cells may have a larger available NADPH pool than the other cell lines, potentially lowering G6PDi-1 sensitivity. Similar, weaker effects are possible in PEX14 KO cells. These may be counterbalanced by the presence of peroxisomal ghosts in PEX14 KO cells, leading to WT-like G6PDi-1 sensitivity. The oxidative stress posed by peroxisomal ghosts may be from accumulated phospholipid intermediates or NADPH-dependent fatty acid biosynthesis processes required for peroxisome membrane biogenesis.

PEX11β KO cells possess fewer, enlarged peroxisomes that may be insufficiently distributed throughout the cytosol to consistently meet metabolic demands. Further, the co-inactivation of PEX11β and PXMP2, a peroxisomal pore protein, perturbs the balance of glutathione and reduced glutathione [27]. It is possible that depleting PEX11β in healthy cells could present redox stress. However, since PEX11β KO cells still contain functional but bottlenecked peroxisomes, this potential stress may be intermittent. Therefore, it may not warrant the systemic metabolic programming likely present in PEX3 KO cells. Instead, PEX11β KO cells might dynamically shift metabolic flux towards lipid storage pathways, as suggested by the accumulation of several TG species. Enlarged peroxisomes in PEX11β-deficient cells may create localized redox sinks, perhaps increasing dependence on the pentose phosphate pathway for NADPH and rendering PEX11β KO cells more vulnerable to G6PD inhibition. Further investigation is necessary to determine the mechanistic basis for G6PD inhibitor sensitivity in PEX11β KO cells, particularly with NADPH/NADP^+^ assays or enzyme kinetics assays. This could explore whether the vulnerability stems from increased NADPH consumption or a diminished capacity for recovery.

Overall, our comparative analysis reveals that peroxisomal dysfunction suggests a metabolic spectrum indicated both by knockout-specific lipid profiles and the cell's compensatory response to oxidative stress. While the total loss of peroxisome biogenesis (PEX3 KO) might trigger metabolic rewiring that confers resistance to certain redox stressors, the disruption of peroxisomal fission (PEX11β KO) may instead encourage metabolic fragility. These findings suggest that potential anticancer therapies targeting peroxisomes could benefit from considering peroxin-specific effects, as biogenesis inhibition may trigger protective adaptations in cancer cells, pending validation in appropriate cancer models.

### 4.1 Limitations

As the data in this study are exploratory, several limitations are present. First, only a single clonal cell line with a healthy background was used for each PEX KO condition, and clone-specific effects are possible. All experiments were also performed purely *in vitro*, and direct measurements of redox balance (NADPH/NADP^+^ ratio, GSH/GSSG ratio) or enzyme activity are lacking, as well as an additional G6PD inhibitor other than G6PDi-1. Pentose phosphate pathway inhibition may also reduce cell viability through other mechanisms not covered in this work. Further, although the untargeted lipidomics approach provided a landscape of the whole cell lysate lipidome, targeted validation and rescue experiments are necessary to make conclusive statements beyond speculation.

## CRediT authorship contribution statement

**Samantha Jeng:** Writing – review & editing, Writing – original draft, Visualization, Validation, Methodology, Investigation, Formal analysis, Data curation, Conceptualization. **Mansha Soni:** Writing – review & editing, Writing – original draft, Visualization, Validation, Methodology, Investigation, Formal analysis, Data curation, Conceptualization. **Vishal C. Kalel:** Writing – review & editing, Formal analysis, Conceptualization. **Wolfgang Schliebs:** Writing – review & editing, Supervision, Project administration, Formal analysis, Conceptualization. **Ralf Erdmann:** Writing – review & editing, Supervision, Resources, Project administration, Funding acquisition. **Frank Schulz:** Writing – review & editing, Supervision, Resources, Project administration, Funding acquisition, Formal analysis.

## Ethics declaration

Written informed consent to take part in the study and to publish the article has been obtained from all participants or their legal representatives. The privacy rights of participants have been observed.

This study included organ or tissue donors. This study includes human biological material and consent was obtained by donors, or their next of kin or legal representatives, for use in this study and for publication of the article. The samples used in this research were not sourced from executed prisoners or prisoners of conscience.

This study was performed in compliance with relevant laws, regulatory frameworks and guidelines where the research took place. Ethics committee approval was not required under relevant laws and institutional guidelines. Commercially available TRex cell lines were used.

## Declaration of the use of AI tools

During the preparation of this work, Google Gemini 3.1 was used for copy editing.

## Funding

The work was supported by the Deutsche Forschungsgemeinschaft (DFG), grant ER178/17-1 and by Ruhr University Bochum through InnovationsFoRUM (Host Microbe Interactions: IF-009N-22 and IF-018N-22) to R.E.

## Declaration of competing interest

The authors declare the following financial interests/personal relationships which may be considered as potential competing interests: Ralf Erdmann reports financial support, administrative support, and article publishing charges were provided by Ruhr University Bochum. Ralf Erdmann reports a relationship with Ruhr University Bochum that includes: employment and funding grants. If there are other authors, they declare that they have no known competing financial interests or personal relationships that could have appeared to influence the work reported in this paper.

## Data Availability

The mass spectrometry dataset was deposited in MassIVE under accession MSV000102129.
