# Supplementary material for: Lipidomic profiling of PEX11β knockout T-REx 293 cells reveals lipid shifts suggesting potential metabolic perturbations
Source: Biochem Biophys Rep. 2026 Sep 17;48:102801. doi: 10.1016/j.bbrep.2026.102801 (PMC13601049; doi:10.1016/j.bbrep.2026.102801)
Supplement: Supplementary file 1 [file mmc2.pdf]

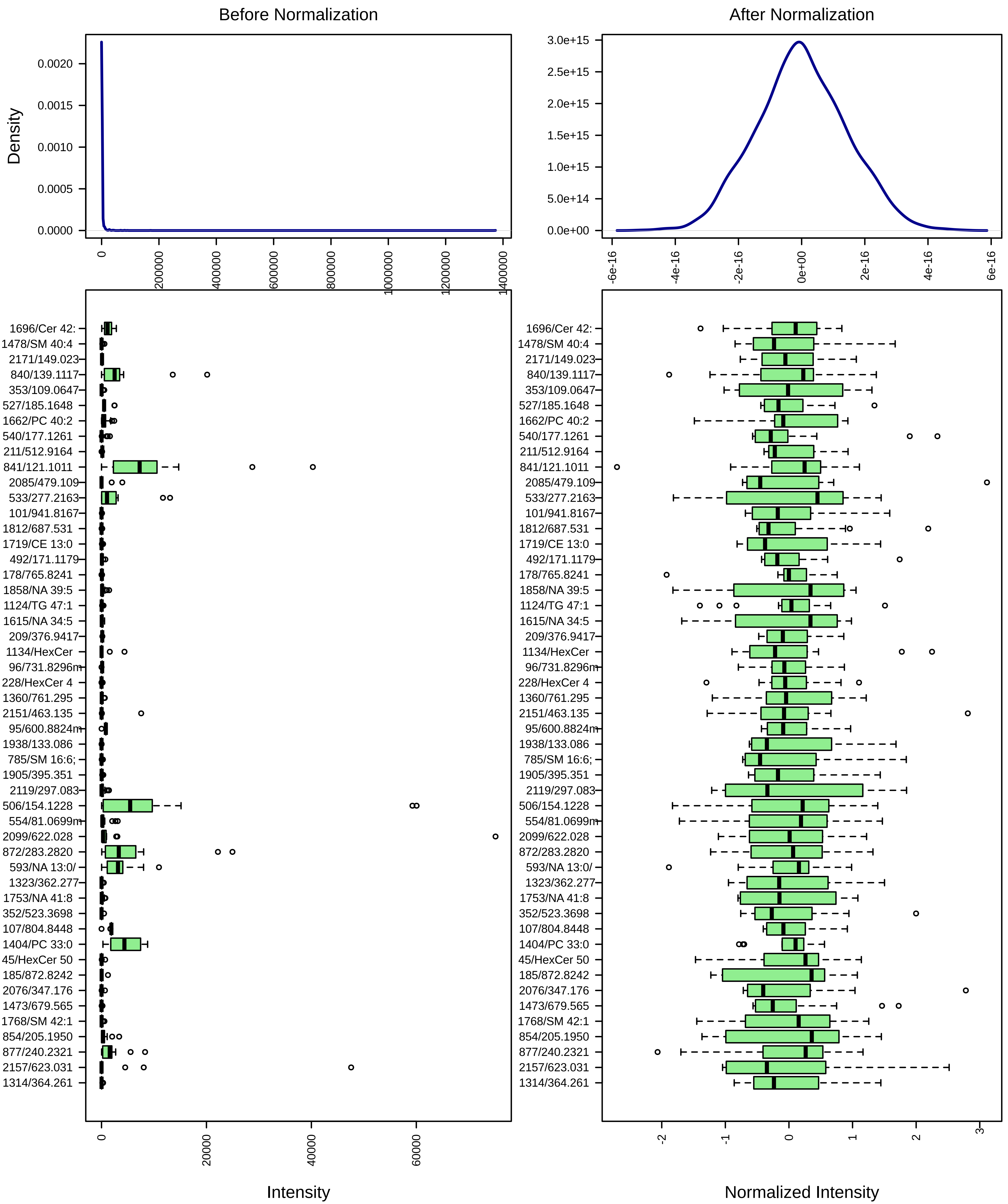
Before Normalization
After Normalization
Density
0.0020
0.0015
0.0010
0.0005
0.0000
0
200000
400000
600000
800000
1000000
1200000
1400000
3.0e+15
2.5e+15
2.0e+15
1.5e+15
1.0e+15
5.0e+14
0.0e+00
-6e-16
-4e-16
-2e-16
0e+00
2e-16
4e-16
6e-16
1696/Cer 42:
1478/SM 40:4
2171/149.023
840/139.1117
353/109.0647
527/185.1648
1662/PC 40:2
540/177.1261
211/512.9164
841/121.1011
2085/479.109
533/277.2163
101/941.8167
1812/687.531
1719/CE 13:0
492/171.1179
178/765.8241
1858/NA 39:5
1124/TG 47:1
1615/NA 34:5
209/376.9417
1134/HexCer
96/731.8296m
228/HexCer 4
1360/761.295
2151/463.135
95/600.8824m
1938/133.086
785/SM 16:6;
1905/395.351
2119/297.083
506/154.1228
554/81.0699m
2099/622.028
872/283.2820
593/NA 13:0/
1323/362.277
1753/NA 41:8
352/523.3698
107/804.8448
1404/PC 33:0
45/HexCer 50
185/872.8242
2076/347.176
1473/679.565
1768/SM 42:1
854/205.1950
877/240.2321
2157/623.031
1314/364.261
0
20000
40000
60000
-2
-1
0
1
2
3
Intensity
Normalized Intensity
