# Supplementary material for: Lipidomic profiling of PEX11β knockout T-REx 293 cells reveals lipid shifts suggesting potential metabolic perturbations
Source: Biochem Biophys Rep. 2026 Sep 17;48:102801. doi: 10.1016/j.bbrep.2026.102801 (PMC13601049; doi:10.1016/j.bbrep.2026.102801)
Supplement: Supplementary file 2 [file mmc1.docx]

**Supplementary Information**

**Parameters for LC-MS acquisition and controls**

Chromatographic separation was performed using a reverse-phase Kinetex C8 Core-shell Column (150 × 2.1 mm, 2.6 µm particle size; Phenomenex). For positive ion mode, the mobile phase consisted of solvent A (60:40 acetonitrile/water, 0.1% formic acid (FA), and 10 mM ammonium formate (AmF)) and solvent B (90:10 isopropanol/acetonitrile, 0.1% FA, and 10 mM AmF). For negative ion mode, the mobile phase consisted of solvent A (60:40 acetonitrile/water, 10 mM ammonium acetate (AmAc) adjusted to pH 8) and solvent B (90:10 isopropanol/acetonitrile, 10 mM AmAc (pH 8)). A multi-step gradient at a flow rate of 0.35 mL/min (reduced to 0.3 mL/min between 22–28 min) was used. The gradient profile for the mobile phase B was: 0–2 min, 30%; 2–5 min, 40%; 5–8 min, 55%; 8–11 min, 66%; 11–14 min, 70%; 14–18 min, 75%; 18–22 min, 95%; 22–28 min, 95%; and 28–33 min, 30%. The autosampler was cooled to 12 °C. The column temperature was maintained at 40 °C. Nitrogen gas was used for nebulization and drying at 10 L/min and 220 °C. Gas pressure was maintained at 2.2 bar, and capillary voltage was set to 4.5 kV for efficient ionization of the analytes. Data-dependent acquisition (DDA) mode was used, where the ten most intense precursor ions exceeding a 480-count threshold (cts) were automatically selected for subsequent fragmentation. Active and smart exclusion filters were enabled.

Mass accuracy calibration was performed with a sodium formate solution injected at the beginning of each measurement. Lock-mass calibration was performed using hexakis(2,2-difluoroethoxy)phosphazene (positive mode: m/z 622.0290; negative mode: m/z 666.0199). To avoid injection order bias, the sample sequence was randomized.

**Sample normalization for LC-MS**

For the normalization of samples for LC-MS, the cell pellets following lipid extraction were resuspended by pipetting and vortexed in 400 μl of 1X SDS-PAGE (Laemmli) sample buffer (containing 0.05 M DTT) and boiled at 95°C for 5 minutes. Of each sample, 10 μl was loaded onto a 12.5% SDS-PAGE gel for electrophoresis and subsequent Western blotting onto a 0.2 μm nitrocellulose membrane. The blots were then stained using Revert™ 700 Total Protein Stain (LI-COR), and detection of the total protein stain was performed in the 700 nm channel using an Odyssey CLx Infrared Imager (LI-COR). Determination of the relative total protein amount per lane was performed using the gel analysis tools in ImageJ (Fiji), selecting entire lanes for analysis and using manual baseline subtraction. LC-MS peak areas were then scaled to the integrated peak areas obtained from the total protein stain calculated in ImageJ.

**Table S1 Parameters for LC-MS data processing in MZmine**

Import MS data

File names: [C:\Data\Hek 293 measurements\17.09.25 Lipid_positive ion mode\WT7_A_BC7_01_5221_modified.mzML

C:\Data\Hek 293 measurements\17.09.25 Lipid_positive ion mode\WT8_A_BD4_01_5226_modified.mzML

Vendor data options: Try vendor centroiding: true

Remove calibrant signals (Thermo): true

Try vendor centroiding: true

Remove calibrant signals (Thermo): true

Apply lockmass on import (Waters): true

Waters MassLynx data import: Native (mzmine as vendor centroiding

recommended)

Advanced import: false

Scan filters: All MS levels

Polarity (Any)

Scan number: <not set>

Base Filtering Integer: <not set>

Retention time: <not set>

Mobility: <not set>

MS level filter: All MS levels

Scan definition:

Polarity: Any

Spectrum type: ANY

Crop MS1 m/z: false (null)

MS1 detector (Advanced): false (Factor of lowest signal)

MS2 detector (Advanced): false (Factor of lowest signal)

Denormalize fragment scans (traps): false

Metadata file: false (null)

Sort and color: true

Spectral library files: []

Mass detection

Raw data files: All raw data files

Evaluation not executed.

Scan filters: MS1

level = 1

Scan number: <not set>

Base Filtering Integer: <not set>

Retention time: [0.0..28.04]

Mobility: <not set>

MS level filter: MS1

level = 1

Scan definition:

Polarity: +

Spectrum type: ANY

Scan types (IMS): All scan types

Denormalize fragment scans (traps): false

Mass detector: Auto

Noise level: 1000.0

Mass detection

Raw data files: All raw data files

Evaluation not executed.

Scan filters: MS2

level = 2

Scan number: <not set>

Base Filtering Integer: <not set>

Retention time: [0.0..28.04]

Mobility: <not set>

MS level filter: MS2

level = 2

Scan definition:

Polarity: +

Spectrum type: ANY

Scan types (IMS): All scan types

Denormalize fragment scans (traps): false

Mass detector: Auto

Noise level: 40.0

Chromatogram builder

Raw data files: All raw data files

Evaluation not executed.

Scan filters: MS1

level = 1

Scan number: <not set>

Base Filtering Integer: <not set>

Retention time: [0.0..28.04]

Mobility: <not set>

MS level filter: MS1

level = 1

Scan definition:

Polarity: +

Spectrum type: ANY

Minimum consecutive scans: 5

Minimum intensity for consecutive scans: 1000.0

Minimum absolute height: 1000.0

m/z tolerance (scan-to-scan): 0.005 m/z or 15.0 ppm

Suffix: chromatograms

Allow single scan chromatograms: {}

13C isotope filter (formerly: isotope grouper)

Feature lists: Feature list name pattern

Evaluation not executed.

Name suffix: deisotoped

m/z tolerance (intra-sample): 0.001 m/z or 5.0 ppm

Retention time tolerance: 0.1 minutes

Mobility tolerance: false (null)

Monotonic shape: false

Maximum charge: 1

Representative isotope: Most intense

Never remove feature with MS2: true

Original feature list: KEEP

Join aligner

Feature lists: Feature list name pattern

Evaluation not executed.

Feature list name: Aligned feature list

m/z tolerance (sample-to-sample): 0.005 m/z or 10.0 ppm

Weight for m/z: 75.0

Retention time tolerance: 0.2 minutes

Weight for RT: 25.0

Mobility tolerance: false (null)

Mobility weight: 1.0

Require same charge state: false

Require same ID: false

Compare isotope pattern: false

Isotope m/z tolerance: 0.001 m/z or 5.0 ppm

Minimum absolute intensity: 0.0

Minimum score: 0.0

Compare spectra similarity: false

Spectral m/z tolerance: 0.001 m/z or 10.0 ppm

MS level: 2

Compare spectra similarity: Weighted cosine similarity

Weights: MassBank (mz^2 * I^0.5)

Minimum cos similarity: 0.7

Handle unmatched signals: KEEP ALL AND MATCH TO ZERO

Original feature list: KEEP

Gap filling

Feature lists: As selected in main window

Evaluation not executed.

Name suffix: gap-filled

Intensity tolerance: 0.2

m/z tolerance (sample-to-sample): 0.001 m/z or 5.0 ppm

Retention time tolerance: 0.2 minutes

Minimum scans (data points): 1

Original feature list: KEEP

Lipid annotation

Feature lists: As selected in main window

Evaluation not executed.

Lipid classes: [FA Free fatty acids

FA Oxidized fatty acids

Side chain parameters: Minimum chain length: 4

Maximum chain length: 26

Minimum chain length: 4

Maximum chain length: 26

Minimum number of DBEs: 0

Maximum number of DBEs: 6

Only search for even chain length: false

m/z tolerance MS1 level:: 0.005 m/z or 10.0 ppm

Search for lipid class specific fragments in MS/MS spectra: true

Merge & select fragment scans: Input scans

Select input scans: Most intense scan across samples

m/z tolerance MS2 level:: 0.005 m/z or 15.0 ppm

Explained intensity [%]:: 0.6

Keep unconfirmed annotations: true

Search for custom lipid class: false

Custom lipid classes: []

Advanced: false

Ions to ignore: []

Export for SIRIUS

Feature lists: As selected in main window

Evaluation not executed.

Filename: C:\Data\Hek 293 measurements\17.09.25 Lipid_positive ion mode\forsirius.mgf

Intensity normalization: IntensityNormalizer[option=No normalization

scientificFormat=true]

Merge & select fragment scans: Merged (preset)

Presets: Single scan: Merged across energies

Merging m/z tolerance: 0.005 m/z or 15.0 ppm

Merge: [Each sample

cross samples]

Intensity merge mode: Maximum value

m/z tolerance: 0.005 m/z or 15.0 ppm

Only rows with annotation: false

Exclude multiple charge: false

Exclude multimers: false

**Table S2 Putative annotations of lipid analytes in PEX KO and WT T-REx293 whole cell lysates.**

| Identification | Elemental Composition | Adduct | m/z (Da) | Error (ppm) | Retention time (min) | Fragments | Identification level (LSI) | Identification level (MSI) |
| --- | --- | --- | --- | --- | --- | --- | --- | --- |
| PC(14:0_14:0) | C_36_H_72_NO_8_P | [M+H]+ | 678.5068 | 0.9350 | 8.97 | 184.0739, 450.2979, 468.3085 | Molecular species level | Level 2 |
| PC(13:0_17:1) | C_38_H_74_NO_8_P | [M+Na]+ | 726.5044 | 0.9046 | 9.38 | 184.0739, 458.2529, 476.3141, 530.2297 | Molecular species level | Level 2 |
| PC(14:0_16:1) | C_38_H_74_NO_8_P | [M+H]+ | 704.5224 | 1.4403 | 9.49 | 184.0739, 468.2957, 494.3589 | Molecular species level | Level 2 |
| PC(14:0_15:0) | C_37_H_74_NO_8_P | [M+Na]+ | 714.5044 | 0.8535 | 9.74 | 184.0739, 464.3138, 468.3085 | Molecular species level | Level 2 |
| PC(15:0_16:0) | C_39_H_78_NO_8_P | [M+H]+ | 720.5537 | 0.5831 | 10.96 | 184.0739, 496.3405, 482.3218, 478.3304, 464.3175 | Molecular species level | Level 2 |
| PC(P-16:0_22:6) | C_46_H_80_NO_7_P | [M+H]+ | 790.5745 | 0.4861 | 10.97 | 184.0739, 480.3344, 550.3190, 279.2710 | Molecular species level | Level 2 |
| PC(P-16:0_14:0) | C_38_H_76_NO_7_P | [M+Na]+ | 712.5237 | 0.9661 | 11.05 | 184.0739, 484.3336, 472.3065 | Molecular species level | Level 2 |
| PC(16:0_24:6) | C_48_H_84_NO_8_P | [M+H]+ | 834.6007 | 1.2142 | 11.12 | 184.0739, 496.3398, 596.3711 | Molecular species level | Level 2 |
| PC(O-16:0_22:6) | C_46_H_82_NO_7_P | [M+H]+ | 792.5901 | 2.3539 | 11.15 | 184.0739, 450.3005, 454.3284, 482.3583 | Molecular species level | Level 2 |
| PC(O-16:0_14:0) | C_38_H_78_NO_7_P | [M+H]+ | 692.5588 | 1.5113 | 11.17 | 184.0739, 454.3284, 436.3256 | Molecular species level | Level 2 |
| PC(P-18:0_22:6) | C_48_H_84_NO_7_P | [M+H]+ | 818.6058 | 1.4123 | 11.15 | 184.0739, 550.4952, 508.3710, 490.3253 | Molecular species level | Level 2 |
| PC(42:7) | C_50_H_86_NO_8_P | [M+H]+ | 860.6149 | 1.6839 | 11.28 | 184.0739 | Species level | Level 3 |
| PC(P-16:0_16:1) | C_40_H_78_NO_7_P | [M+H]+ | 716.5588 | 1.2920 | 11.34 | 184.0739, 476.3105, 480.3446 | Molecular species level | Level 2 |
| PC(P-16:0_16:0) | C_40_H_80_NO_7_P | [M+H]+ | 718.5745 | 1.4016 | 11.48 | 184.0739, 478.3291, 480.3448 | Molecular species level | Level 2 |
| PC(O-16:0_15:0) | C_39_H_80_NO_7_P | [M+H]+ | 706.5745 | 0.5672 | 11.61 | 184.0739, 482.3618, 466.3253 | Molecular species level | Level 2 |
| PC(O-16:0_17:1) | C_41_H_82_NO_7_P | [M+H]+ | 732.5901 | 2.2450 | 11.85 | 184.0739, 480.3424, 492.3308 | Molecular species level | Level 2 |
| PC(44:7) | C_52_H_90_NO_8_P | [M+H]+ | 888.6474 | 0.3554 | 12.03 | 184.0739 | Species level | Level 3 |
| PC(P-16:0_16:0) | C_40_H_80_NO_7_P | [M+H]+ | 718.5745 | 1.1491 | 12.05 | 184.0739, 478.3286, 480.3475 | Molecular species level | Level 2 |
| PC(20:5_22:1) | C_50_H_88_NO_8_P | [M+H]+ | 862.6320 | 1.5219 | 12.04 | 184.0739, 524.3582, 542.3199, 560.3347 | Molecular species level | Level 2 |
| SM(34:0;3O) | C_39_H_81_N_2_O_7_P | [M+H]+ | 721.5854 | 9.9310 | 12.13 | 184.0739, 538.5338, 703.6247 | Species level | Level 3 |
| PC(O-16:0_16:0) | C_40_H_82_NO_7_P | [M+H]+ | 720.5901 | 1.5654 | 12.15 | 184.0739, 478.3306, 482.3596 | Molecular species level | Level 2 |
| PC(P-16:0_18:1) | C_42_H_82_NO_7_P | [M+H]+ | 744.5901 | 1.7761 | 12.21 | 184.0739, 462.3399, 480.3442, 504.3452 | Molecular species level | Level 2 |
| SM(36:1;3O) | C_41_H_83_N_2_O_7_P | [M+H]+ | 747.6010 | 9.7093 | 12.32 | 184.0739, 266.2533,  564.5385, 729.6358 | Species level | Level 3 |
| PC(O-16:0_18:1) | C_42_H_84_NO_7_P | [M+H]+ | 746.6058 | 2.2820 | 12.31 | 184.0739, 504.3428, 482.3590, 265.2510 | Molecular species level | Level 2 |
| PC(O-18:0_18:2) | C_44_H_86_NO_7_P | [M+H]+ | 772.6214 | 2.7112 | 12.47 | 184.0739, 502.3251, 510.3968 | Molecular species level | Level 2 |
| PC(O-18:1_20:4) | C_46_H_84_NO_7_P | [M+H]+ | 794.6058 | 0.6524 | 12.50 | 184.0739, 508.3830, 526.3311 | Molecular species level | Level 2 |
| PC(24:1_20:5) | C_52_H_92_NO_8_P | [M+H]+ | 890.6615 | 2.0311 | 12.65 | 184.0739, 542.3657 | Molecular species level | Level 2 |
| PC(O-17:0_18:1) | C_43_H_86_NO_7_P | [M+H]+ | 760.6214 | 5.8493 | 12.75 | 184.0739, 496.3375, 504.3326 | Molecular species level | Level 2 |
| SM(36:0;3O) | C_41_H_85_N_2_O_7_P | [M+H]+ | 749.6167 | 9.8731 | 13.06 | 184.0739, 266.2598, 566.6406 | Species level | Level 3 |
| PC(O-18:0_16:0) | C_42_H_86_NO_7_P | [M+H]+ | 748.6214 | 2.5982 | 13.07 | 184.0739, 478.3313, 510.3906 | Molecular species level | Level 2 |
| PC(P-18:0_18:1) | C_44_H_86_NO_7_P | [M+H]+ | 772.6214 | 2.3156 | 13.09 | 184.0739, 265.2502, 307.2917, 490.3603, 506.3582, 508.3885 | Molecular species level | Level 2 |
| PC(O-18:0_18:1) | C_44_H_88_NO_7_P | [M+H]+ | 774.6371 | 2.3207 | 13.22 | 184.0739, 504.3432, 510.3913 | Molecular species level | Level 2 |
| SM(38:1;3O) | C_43_H_87_N_2_O_7_P | [M+H]+ | 775.6323 | 8.2865 | 13.22 | 184.0739 | Species level | Level 3 |
| DG(32:0) | C_35_H_68_O_5_ | [M+Na]+ | 591.4958 | 1.4001 | 13.51 | 95.0852, 85.1017, 103.0576 | Species level | Level 3 |
| DG(24:5_18:1) | C_45_H_76_O_5_ | [M+Na]+ | 719.5584 | 2.1858 | 13.87 | 95.0806,  341.2978, 265.2499, 245.2394 | Molecular species level | Level 2 |
| DG(33:0) | C_36_H_70_O_5_ | [M+Na]+ | 605.5115 | 0.9707 | 13.90 | 95.0852, 85.1017, 103.0576 | Species level | Level 3 |
| PC(O-20:0_18:1) | C_46_H_92_NO_7_P | [M+H]+ | 802.6684 | 0.0763 | 13.98 | 184.0739, 265.2727, 504.3453, 538.4176 | Molecular species level | Level 2 |
| PC(16:0_24:1) | C_48_H_94_NO_8_P | [M+H]+ | 844.678 | 2.1174 | 14.13 | 184.0739, 496.3295, 588.4263, 606.4559 | Molecular species level | Level 2 |
| PC(18:1_24:1) | C_50_H_96_NO_8_P | [M+H]+ | 870.6946 | 0.6693 | 14.21 | 184.0739, 504.3478, 522.4224, 588.4394 | Molecular species level | Level 2 |
| DG O-37:7 | C_40_H_65_O_3_ | [M+H-H_2_O]+ | 593.4906 | 3.7068 | 14.21 | 77.0387, 95.0832, 310.3104 | Species level | Level 3 |
| DG(18:1_16:0) | C_37_H_70_O_5_ | [M+H-H_2_O]+ | 577.5163 | 4.67 | 14.27 | 95.0855, 265.2500, 239.2385 | Molecular species level | Level 2 |
| PC(O-16:0_24:1) | C_48_H_96_NO_7_P | [M+H]+ | 830.6997 | 1.0051 | 14.68 | 184.0739, 480.3522, 590.4413 | Molecular species level | Level 2 |
| PC(16:0_24:0) | C_48_H_96_NO_8_P | [M+H]+ | 846.6946 | 1.2277 | 14.81 | 184.0739, 478.3229, 590.4456 | Molecular species level | Level 2 |
| PC(18:1_24:0) | C_50_H_98_NO_8_P | [M+H]+ | 872.7102 | 1.0046 | 14.84 | 184.0739, 504.3346, 590.4464, 608.4641 | Molecular species level | Level 2 |
| PC(22:1_22:1) | C_52_H_100_NO_8_P | [M+H]+ | 898.7259 | 1.1010 | 14.89 | 184.0739, 560.4346, 578.7891 | Molecular species level | Level 2 |
| MG(20:0) | C_23_H_46_O_4_ | [M+H-H_2_O]+ | 369.3361 | 0.5415 | 14.98 | 73.0470, 75.0242, 295.2956, 313.3085 | Molecular species level | Level 2 |
| PC(16:0_26:0) | C_50_H_100_NO_8_P | [M+H]+ | 874.7259 | 1.5318 | 15.52 | 184.0739, 496.3412, 636.4918 | Molecular species level | Level 2 |
| PC(20:0_24:1) | C_52_H_102_NO_8_P | [M+H]+ | 900.7415 | 1.7493 | 15.59 | 184.0739, 552.4026, 588.3788 | Molecular species level | Level 2 |
| DG(40:1) | C_43_H_82_O_5_ | [M+Na]+ | 701.6054 | 2.1330 | 15.67 | 95.0852, 85.1017, 103.0576 | Species level | Level 3 |
| Cer(40:3;3O) | C_40_H_75_NO_4_ | [M+H]+ | 634.5768 | 6.3657 | 15.67 | 264.2685, 282.2791 | Molecular species level | Level 2 |
| MG(24:1) | C_27_H_52_O_4_ | [M+H-H_2_O]+ | 423.3830 | 2.1257 | 15.68 | 73.0471, 349.3439 | Molecular species level | Level 2 |
| MG(24:0) | C_27_H_54_O_4_ | [M+H-H_2_O]+ | 425.3987 | 1.8895 | 16.38 | 73.0471, 351.3654 369.3708 | Molecular species level | Level 2 |
| DG(40:0) | C_43_H_84_O_5_ | [M+H-H_2_O]+ | 663.6273 | 2.8630 | 16.39 | 95.0852, 85.1017, 103.0576 | Species level | Level 3 |
| MG(26:1) | C_29_H_56_O_4_ | [M+H-H_2_O]+ | 451.4141 | 0.8864 | 16.40 | 73.0262, 377.3742 | Molecular species level | Level 2 |
| DG(42:1) | C_45_H_86_O_5_ | [M+H-H_2_O]+ | 689.6439 | 0.4350 | 16.39 | 95.0852, 85.1017, 103.0576 | Species level | Level 3 |
| DG(20:0_20:1) | C_43_H_82_O_5_ | [M+H-H_2_O]+ | 661.6089 | 6.9526 | 16.46 | 495.2666, 383.1395, 293.2273, 349.2447 | Molecular species level | Level 2 |
| DG(42:0) | C_45_H_88_O_5_ | [M+H-H_2_O]+ | 691.6597 | 1.1566 | 17.15 | 95.0852, 85.1017, 103.0576 | Species level | Level 3 |
| DG(24:0_18:1) | C_45_H_86_O_5_ | [M+H-H_2_O]+ | 689.6405 | 6.235 | 17.20 | 75.0446, 95.0854, 339.3109, 425.4419 | Molecular species level | Level 2 |
| CerPE(34:1;O2) | C_36_H_74_N_2_O_6_P | [M+H]+ | 661.5319 | 6.046 | 17.82 | 80.9476, 95.0836, 96.0775, 369.3505 | Species level | Level 3 |
| DG(16:0_16:1) | C_35_H_66_O_5_ | [M+H-H_2_O]+ | 549.4870 | 2.3658 | 18.53 | 95.0855,  237.2208, 239.2363, 311.2569, 313.2716 | Molecular species level | Level 2 |
| TG(14:0_O-18:0_16:0) | C_51_H_100_O_5_ | [M+Na]+ | 815.7462 | 2.1160 | 20.51 | 537.5228, 559.5147, 565.5696 | Molecular species level | Level 2 |
| TG(16:0_16:1_O-18:0) | C_51_H_100_O_5_ | [M+Na]+ | 815.7462 | 1.0471 | 20.58 | 239.2326, 563.5300, 587.5302 | Molecular species level | Level 2 |
| PE(16:0_16:1) | C_37_H_72_NO_8_P | [M-H]- | 688.4913 | 1.4414 | 10.89 | 140.0083, 253.2155, 255.2306 | Molecular species level | Level 2 |
| PE(16:0_18:1) | C_39_H_76_NO_8_P | [M-H]- | 716.5221 | 2.0642 | 11.86 | 140.0083, 281.2483, 255.2342 | Molecular species level | Level 2 |
| PE(P-16:0_22:6) | C_43_H_74_NO_7_P | [M-H]- | 746.5120 | 1.2913 | 11.16 | 140.0083, 327.2328 | Molecular species level | Level 2 |
| PC(14:0_14:0) | C_36_H_72_NO_8_P | [M+CH_3_COO]- | 736.5127 | 0.9790 | 8.89 | 168.0403, 227.2028 | Molecular species level | Level 2 |

**Principal component analysis (PCA) of the LC-MS dataset in higher dimensions**

**Figure S1 Three-dimensional (3D) principal component analysis (PCA) plot.**

When the third principal component (PC3, 11.3% of all variance) is applied to the LC-MS dataset already separated by principal components in two dimensions, the separation between the clusters by cell line improves slightly.

**Figure S2 Principal component analysis (PCA) pairwise plot.**

Based on this pairwise plot of principal components (PCs), complete separation between the clustered cell line groups is achieved already with PC1 and PC2. While some additional separation can be achieved by including PC3 based on the 3D PCA plot (Figure S1), as well as additional separation between the PEX3 and PEX14 KO clusters by including PC4 in addition to PC2, the separation achieved by PC1 and PC2 is sufficient. This is further supported by the evidence that PEX3 and PEX14 KO cells have highly similar defects within their lipidomes.

**Pairwise comparisons between differentially expressed lipid analytes in PEX KO T-REx 293 cell lipidomes**

**Figure S3 Heatmap of differentially expressed lipid analytes between PEX3 and PEX11β KO T-REx 293 cells.**

PEX3 KO cells are primarily enriched in very-long-chain fatty acids (VLCFAs) compared to PEX11β KO cells, which are higher in ether and plasmalogen phosphatidylcholines, as well as the ceramide Cer(40:3;3O).

**Figure S4 Heatmap of differentially expressed lipid analytes between PEX3 and PEX14 KO T-REx 293 cells.**

Here, the lower-chain-length phosphatidylcholines and ether phosphatidylcholines (~C13 to C17) may represent constituents of the peroxisomal membrane, as PEX3 KO cells lack peroxisomal membranes, while PEX14 KO cells have intact peroxisomal membranes but disrupted import, resulting in peroxisomal ghosts.

**Figure S5 Heatmap of differentially expressed lipid analytes between PEX11β and PEX14 KO T-REx 293 cells.**

PEX14 KO cells are primarily enriched in very-long-chain fatty acids (VLCFAs) compared to PEX11β KO cells, which are higher in ether and plasmalogen phosphatidylcholines, as well as the ceramide Cer(40:3;3O). Here, the effects are similar to those observed in the PEX3 KO-PEX11β KO pairwise comparison (Figure S1), as the lipid pathways impaired by disrupted peroxisomal import (PEX14 KO) are shared by those impaired by fully absent peroxisomal membranes (PEX3 KO).

**Figure S6 Volcano plots of differentially expressed lipid analytes in pairwise comparisons of PEX KO T-REx 293 cells.**

Here, the most differentially expressed lipid analytes are displayed in volcano plots in pairwise comparisons between the PEX KO cell lines. Among the different pairwise comparisons, the greatest difference in lipid composition is observed in **(a)** PEX3 KO and PEX11β KO cells. Between **(b)** PEX3 KO and PEX14 KO cells, the lipidomes differ by fewer lipid analytes, although several analytes are distinctly lower in PEX3 KO cells than PEX14 KO cells. The lipidomes of **(c)** PEX11β KO and PEX14 KO cells differ in a similar but less pronounced manner than those of **(a)** PEX3 KO and PEX11β KO cells.

**Lipid ontology (LION) analysis of relevant metabolic pathways in PEX KO T-REx293 cells**

Further details on the LION terms and specific lipid identifiers used for enrichment analysis can be found in Supplementary Information 5.

**Figure S7 Lipid ontology (LION) enrichment analysis of PEX3 KO vs. WT T-REx 293 cells.**

In this LION analysis of PEX3 KO T-REx 293 cells compared to a wild-type background, the accumulation of very-long-chain fatty acids (“fatty acid with 24 carbons”) may contribute to lipid membrane sluggishness and thickness (“very low lateral diffusion”, “very high/above average bilayer thickness”). Interestingly, the analysis also predicts the enrichment of “lipid-mediated signaling” pathways, suggesting that the absence of PEX3 results in abnormal lipid signaling within the cell.

**Figure S8 Lipid ontology (LION) enrichment analysis of PEX11β KO vs. WT T-REx 293 cells.**

The LION analysis of PEX11β KO pathways compared to the wild-type background in T-REx 293 cells suggests little significant change. Although some ceramide species are observed as being very highly enriched when the PEX11β KO lipidome is compared to those of all other cell lines, it appears that these few species do not indicate a broad rerouting of ceramide pathways.

**Figure S9 Lipid ontology (LION) enrichment analysis of PEX14 KO vs. WT T-REx 293 cells.**

Strikingly, the “diacylglycerophosphocholine [GPO101]” pathway shows strong enrichment in this LION analysis of PEX14 KO cells against a wild-type T-REx 293 background. As this is the most abundant type of phospholipid found in mammalian cell membranes, this suggests that membrane production or turnover is more prominently featured in PEX14 KO cells. Other enriched pathways include those similar to the ones found in the PEX3 KO LION analysis (Figure S7) and to a similar degree, suggesting that these pathways are most impaired by the lack of functional peroxisomal matrix import.

**Validation of PEX KO T-REx 293 cell lines**

**Sequences of PEX11β exon 2 in WT and all three KO alleles**

**WT:**

GG**CCG**CCCAGTATGCTTGCTCTCTTCTTGGCCATGCGCTGCAGAGGCATGGAGCCAGTCCTGAGTTACAGAAACAGATTCGACAAC**TGG**AGAGCCACCTGAGCCTTGGAAGAAAGC

**PEX11β KO allele 1 (+1):** GGGCCGCCCA**A**GTATGCTTGCTCTCTTCTTGGCCATGCGCTGCAGAGGCATGGAGCCAGTCC**TGA**GTTACAGAAACAGATTCGACAACTGGAGAGCCACCTGAGCCTTGGAAGAAAGCGTAAGTAACTCT

**PEX11β KO allele 2 (-1, -3):**

GGGCCGCCCA_TATGCTTGCTCTCTTCTTGGCCATGCGCTGCAGAGGCATGGAGCCAGTCCTGAGTTACAGAAACAGATTCGAC___TGGAGAGCCACC**TGA**GCCTTGGAAGAAAGCGTAAGTAACTCT

**PEX11β KO allele 3 (-29, -87):**

**_______________**TTGCCTGCCCTCTCTTCTTGGCCATGCGCTGCAGAGGCATGGAGCCAGTCC**TGA**GTTACA**__________________________________________________**

PAM sequences are indicated in the WT sequence in the bolded underlined text. Insertions are indicated in bolded underlined green, and deletions are shown as red underscores. The numbers in parentheses indicate the sizes of the insertions and deletions in each allele. Sanger sequencing was performed by Microsynth SeqLab (Göttingen, Germany). All three PEX11β KO alleles are predicted to result in premature stop codons, leading to loss-of-function (indicated by bolded underlined purple text).

**Figure S10 Validation of PEX11β knockout by functional complementation**

**(a)** Transfection of T-REx293 WT and PEX11β KO cells with a PEX11β-expressing pIRES2 eGFP-PTS1 vector allowed for the quantification of peroxisomes. **(b)** Quantification of peroxisomes in T-REx293 WT (n=79) and PEX11β KO (n=80) revealed that the knockout had drastically fewer (p<0.0001) peroxisomes, at about 86 peroxisomes per cell from 207 per cell in the WT. This was almost completely restored to 167 by complementation with the PEX11β-expressing vector (p<0.0001). **(c)** Similarly, the average volume of the peroxisomes segmented in 3D with ImageJ software was 0.06545 µm^3^ in the WT (n=2899 peroxisomes), much higher in the PEX11β KO (0.1037 µm^3^, n=1207), and nearly restored for the PEX11β-complemented PEX11β KO cells (0.07615 µm^3^, n=2654). Thus, the PEX11β knockout cell line was validated by restoration of peroxisome morphology in PEX11β-complemented PEX11β KO cells.

**Determination of peroxisomal volume from fluorescence microscope images**

Peroxisomal volume was determined using ImageJ with the Bio-Formats Importer, 3DSuite, and MorphoLib plugins. First, the rabbit α-PMP70-stained red (secondary antibody: goat α-rabbit Alexa Fluor 594) channel was isolated, and a rolling ball (radius=5.0 pixels) subtraction was performed to remove large artifacts. Next, the 3D Maxima Finder was used from 3DSuite, and the threshold was adjusted to a value between 1500 and 3000 before Image > Adjust > Threshold to a value of roughly 200 above the automatically applied threshold. Then, watershed segmentation was used from 3DSuite to perform 3D segmentation of the peroxisomes before selecting “Analyze Regions 3D” from MorphoLibJ to determine individual peroxisome volumes. Peroxisomal number and volume were compared using one-way ANOVA using default settings from GraphPad Prism 8.

**PEX3 KO and PEX14 KO cell line validation**

Validation of PEX3 and PEX14 KO cells was performed by Ott et al. [1] in previous work by the lab comparing other in-house PEX KO mutant cell lines. Similarly, complementation of PEX3- and PEX14-deficient cells with a pIRES2 eGFP-SKL expression vector demonstrated successful knockout and rescue both via fluorescence microscopy and Western blot. The same clonal PEX3 and PEX14 KO cell lines were used in the present work.

*Reference:*

[1]	J. Ott, J. Sehr, N. Schmidt, W. Schliebs, and R. Erdmann, “Comparison of human PEX knockout cell lines suggests a dual role of PEX1 in peroxisome biogenesis,” *Biol. Chem.*, vol. 404, no. 2–3, pp. 209–219, Feb. 2023, doi: 10.1515/hsz-2022-0223.
